# T-bet regulates differentiation of forkhead box protein 3^+^ regulatory T cells in programmed cell death-1-deficient mice

**DOI:** 10.1111/cei.12455

**Published:** 2015-01-02

**Authors:** M Tahara, Y Kondo, M Yokosawa, H Tsuboi, S Takahashi, S Shibayama, I Matsumoto, T Sumida

**Affiliations:** * Department of Internal Medicine, Faculty of Medicine, University of TsukubaTsukuba, Ibaraki, Japan; § Department of Anatomy and Embryology, Faculty of Medicine, University of TsukubaTsukuba, Ibaraki, Japan; ‡ Laboratory Animal Resource Center, University of TsukubaTsukuba, Ibaraki, Japan; § Tsukuba Institute ONO Pharmaceutical Co., LTD.Tsukuba, Ibaraki, Japan

**Keywords:** FoxP3^+^ regulatory T cells, helper T cell subsets, programmed cell death-1, T cell tolerance, Th1 cells

## Abstract

Programmed cell death-1 (PD-1) plays an important role in peripheral T cell tolerance, but whether or not it affects the differentiation of helper T cell subsets remains elusive. Here we describe the importance of PD-1 in the control of T helper type 1 (Th1) cell activation and development of forkhead box protein 3 (FoxP3^+^) regulatory T cells (T_regs_). PD-1-deficient T cell-specific T-bet transgenic (P/T) mice showed growth retardation, and the majority died within 10 weeks. P/T mice showed T-bet over-expression, increased interferon (IFN)-γ production by CD4^+^ T cells and significantly low FoxP3^+^ T_reg_ cell percentage. P/T mice developed systemic inflammation, which was probably induced by augmented Th1 response and low FoxP3^+^ T_reg_ count. The study identified a unique, previously undescribed role for PD-1 in Th1 and T_reg_ differentiation, with potential implication in the development of Th1 cell-targeted therapy.

## Introduction

Naive CD4^+^ T cells can differentiate into at least four subsets; T helper type 1 (Th1), Th2, Th17 and regulatory T cells (T_regs_) 1–4. Th1 cells predominantly produce proinflammatory cytokines [interferon (IFN)-γ and interleukin (IL)-2] and are necessary for the elimination and control of many intracellular pathogens 5. The transcription factor T-bet is thought to be both necessary and sufficient for Th1 cell differentiation from naive T cells 6–8, and directly controls IFN-γ gene expression. Although Th1 responses are essential for the control and prevention of diseases, activation of Th1 cells in response to self-antigen or innocuous antigens (derived from food, airborne particulate material or from gut commensals) is thought to be involved in the pathogenesis of autoimmune diseases, such as multiple sclerosis 9, rheumatoid arthritis 10, systemic lupus erythematosus 11, intestinal inflammation 12–14, type 1 diabetes 15, contact dermatitis and contact hypersensitivity (CHS) 16–18. Therefore, the mechanism involved in the regulation of Th1 cell activation is important for understanding the pathogenesis of diseases induced by the abnormal Th1 immune response.

To examine the regulation of Th1 cell activation, we focused upon programmed cell death-1 (PD-1), a type 1 transmembrane protein composed of immunoglobulin superfamily domain, a transmembrane domain and an intracellular domain containing an immune-receptor tyrosine-based inhibitory motif (ITIM) and an immune-receptor tyrosine-based inhibitory switch motif (ITSM) 19,20. Although PD-1 was identified initially as a gene that induced programmed cell death 21, subsequent experiments indicated that PD-1 expression is induced in activated T cells, B cells and monocytes, and it is now clear that the major function of PD-1 is to attenuate the immune response 21–26. PD-1/PD-L1 interactions are considered to inhibit T cell receptor (TCR) signalling 27, resulting in the suppression of immune response induced by interaction with dendritic cells (DC) 28. Although recent studies showed that T-bet is directly bound to a T-bet regulatory region upstream of the PDCD1 gene and represses the expression of PD-1 29,30, it remains to be determined whether or not PD-1 specifically affects the expression of T-bet and differentiation of Th1 cells.

The present study was designed to further investigate the role of PD-1 on the differentiation and function of Th1 cells. For this purpose, we crossed PD-1 knock-out (KO) mice and T-bet transgenic (T-bet Tg) mice under the control of the CD2 promoter. Surprisingly, PD-1 KO × T-bet Tg (P/T) mice showed growth retardation, and the majority of P/T mice died within 10 weeks. Mononuclear cell infiltration was observed in the liver, pancreas and intestine, and immunohistochemical analysis identified infiltrating cells in liver as CD3^+^CD4^+^ T cells. T-bet expression and IFN-γ production were increased appreciably in splenic CD4^+^ T cells in P/T mice. Moreover, forkhead box protein 3 (FoxP)3^+^CD4^+^ T cells were decreased significantly in the spleen and thymus in P/T mice compared with wild-type (WT), PD-1 KO and T-bet Tg mice. *In-vitro* experiments showed no induction of FoxP3 expression on CD4^+^ T cells from P/T mice under T_reg_ differentiation conditions with transforming growth factor (TGF)-β. Recombination activating gene 2 (Rag-2) KO mice transferred with splenocytes of P/T mice showed body weight loss, together with inflammatory cell infiltration in liver, pancreas, intestine and skin, similar to P/T mice. Co-transfer of CD4^+^ CD25^−^ T cells of P/T mice with CD4^+^CD25^+^ cells isolated from WT mice attenuated infiltration of mononuclear cells in liver, pancreas, intestine and skin in Rag-2 KO mice. The results indicated that PD-1 deficiency in T-bet Tg mice caused systemic inflammation, resulting in a short life span, which was due probably to an augmented Th1 response and reduction of FoxP3^+^ CD4^+^ regulatory T cells. The findings suggested that the control of PD-1 signal transduction could be a new therapeutic approach for inflammatory disorders induced by the Th1 immune response.

## Materials and methods

### Mice

CD2 T-bet transgenic mice 31,32 were prepared by back-crossing mice on the C57BL/6 background. PD-1 KO mice were obtained from the Institute of Physical and Chemical Research (RIKEN) (Wako, Japan) 23,24. C57BL/6 (WT) mice were used as negative control. All mice were maintained under specific pathogen-free conditions. Experiments were conducted following the approval of the University of Tsukuba animal ethics committee (authorization no. 13–277). In order to minimize suffering, if mice were found in a moribund state as defined by the University of Tsukuba animal ethics committee they were anaesthetized with 30% isoflurane prior to cervical dislocation. The condition of the mice was monitored once a day.

### Skin phenotype

Dermatitis is evaluated visually, as reported previously by Ishizaki . 31, which is characterized by swollen, flaky and scaly skin in regions without body hair.

### Body and spleen weight

Body weight was measured from mice at 5 weeks of age, and spleen weight was measured from 6 to 10 weeks of age using an electric balance.

### Histopathological analysis

The kidney, heart, spleen, lung, liver, pancreas, salivary gland, lacrimal gland, intestine, mesenteric lymph nodes and ear skin were harvested, fixed with 10% formalin in phosphate-buffered saline (PBS) and embedded in paraffin. Sections were stained with haematoxylin and eosin (H&E) using standard methods.

### Immunohistochemistry

The following anti-mouse primary antibodies were used for immunohistochemical analysis: Alexa Fluor 647-labelled B220 (Invitrogen, Carlsbad, CA, USA), Alexa Fluor 647-labelled CD4 (Invitrogen), unconjugated anti-CD3ε (Biolegend, San Diego, CA, USA) and anti-CD8 (Biolegend). The following secondary antibodies were used: Alexa Fluor 488-labelled anti-hamster IgG (Biolegend) and Alexa Fluor 546-labelled anti-rat IgG (Invitrogen). All antibodies were diluted in 1% bovine serum albumin (BSA) in PBS before application to the tissue sections. The liver was embedded in optimal cutting temperature (OCT) compound (Sakura, Torrance, CA, USA) and snap-frozen. Next, 4–5-μm-thick sections were air-dried, fixed with ice-cold acetone and rehydrated in PBS. After washing with 0·05% Tween 20 in PBS, blocking buffer (1% BSA in PBS) was added, and the sections were incubated for 30 min at room temperature. After washing, the primary antibody was added, followed by incubation overnight at 4°C. After washing, the secondary antibody was added, followed by incubation for 30 min at room temperature. After washing, 4′,6-diamidino-2-phenylindole (DAPI) in 1% BSA in PBS was added, and the preparation was incubated for 5 min at room temperature. After washing, fluorescent mounting medium (Dako, Glostrup, Denmark) was added and sections were analysed by a fluorescence microscope (BZ-9000; Keyence or FV10i; Olympus, Tokyo, Japan).

### Measurement of serum autoantibodies and biochemical analysis of sera

Sera were obtained from mice at 6–10 weeks of age. Serum levels of glutamic oxaloacetic transaminase (GOT), glutamic pyruvic transaminase (GPT) and amylase were measured by the DRI-CHEM protocol (Fujifilm, Tokyo, Japan). The sample was added to a specified slide and analysed.

### Surface and intracellular staining and FACS analysis

GolgiStop (BD PharMingen, San Diego, CA, USA), phorbol myristate acetate (PMA) and ionomycin were added during the last 4 h of each culture. A T_reg_ staining kit (eBioscience) was used to stain FoxP3 in spleen and thymus cells according to the protocol provided by the manufacturer using anti-forkhead box protein 3 (FoxP3)-PE (eBioscience), anti-T-bet-PE (eBioscience) and anti-IFN-γ-APC (BD Pharmingen). Samples were analysed with fluorescence activated cell sorter (FACS)Calibur flow cytometer (Becton Dickinson, Mountain View, CA, USA), and data were analysed with FlowJo software (Tree Star, Ashland, OR, USA).

### *In-vitro* CD4^+^ T cell cultures

Spleen CD4^+^ cells were isolated by positive selection using the magnetic affinity cell sorting (MACS) system with anti-CD4 monoclonal antibody (mAb) (Miltenyi Biotec, Bergisch Gladbach, Germany). CD4^+^ T cells (5 × 10^5^ cells/well) were cultured for 48 h in RPMI-1640 medium (Sigma-Aldrich, St Louis, MO, USA) containing 10% fetal bovine serum (FBS), 100 units/ml of penicillin and 100 μg/ml of streptomycin with 1 μg/ml soluble anti-CD3ε mAb (eBioscience) and 1 μg/ml soluble anti-CD28 mAb (BioLegend).

### Measurement of IFN-γ and IL-17 concentrations

Culture supernatants were collected from CD4^+^ cell cultures. IFN-γ and IL-17 were measured using the Duoset enzyme-linked immunosorbent assay (ELISA) kit (R&D Diagnostic, Minneapolis, MN, USA), according to the protocol provided by the manufacturer. Diluted capture antibody in PBS was coated overnight on 96-well plates (Nunc Maxisorp; Nalge Nunc International, Roskilde, Denmark) at room temperature. After washing with wash buffer (0·05% Tween 20 in PBS, pH 7·2–7·4), 300 μl of blocking buffer (1% BSA in PBS with 0·05% NaN_3_) was added, followed by further incubation for 2 h at room temperature. After washing, 100 μl of the culture supernatant was added, and the plates were incubated for 2 h at room temperature. After washing, 100 μl of detection antibody in reagent diluent [special IFN-γ; 0·1% BSA, 0·05% Tween 20 in Tris-buffered saline (20 mM Trizma base, 150 mM NaCl), pH 7·2–7·4, 0·2 μm filtered] (special IL-17; 1% BSA in PBS, pH 7·2–7·4, 0·2 μm filtered) was added and incubated for 2 h at room temperature. After washing, 100 μl streptavidin–horseradish peroxidase (HRP) in reagent diluent was added, and the preparation was incubated further for 20 min at room temperature under darkness. After washing, 100 μl of 3,3′,5,5′-tetramethylbenzidine (TMB) was added, followed by incubation for 20 min at room temperature in darkness. Finally, 50 μl of stop solution (2 N H_2_SO_4_) was added, and the optical density was read at 450 nm using a microplate reader.

### Quantitative analysis of cytokine in sera

Cytometric bead array (CBA mouse inflammation kit; BD PharMingen) was used to quantify IFN-γ, tumour necrosis factor (TNF)-α, IL-12p70, monocyte chemoattractant protein-1 (MCP-1) and IL-6 in sera. For more information, cytokine beads in solutions A1, A2, A3, A4, A5 and A6 provided in the kit were vortexed and added together in the tube. The mixture was added at 50 μl per sample. Wash buffer (1 ml) was added to each sample and spun at 180 xg 15°C for 5 min. After washing, beads were resuspended with 300 μl of wash buffer. Samples were vortexed before analysis on a FACScalibur (Becton Dickinson) with CellQuest and CBA analysis software version 1·4.2 for Microsoft Office Excell 2000.

### *In-vitro* induction of FoxP3^+^ T_reg_ differentiation

CD4^+^ cells in spleen were isolated by positive selection using MACS with anti-CD4 mAb (Miltenyi Biotec). The CD4^+^ T cells were stimulated for 5 days with plate-bound anti-CD3 (1 μg/ml) and soluble anti-CD28 (1 μg/ml) in the presence of recombinant human (rh)TGF-β1 (5 ng/ml; R&D Diagnostic), anti-IFN-γ antibody (10 μg/ml; BioLegend) and IL-2 (100 U/ml; MBL, Nagoya, Japan) in complete RPMI-1640 medium containing 10% FBS, 100 units/ml of penicillin and 100 μg/ml of streptomycin.

### *In-vitro* T_reg_ suppression assays

CD4^+^CD25^+^ cells and CD4^+^CD25^−^ cells in spleen were isolated by positive and negative selection using MACS with a CD4^+^CD25^+^ regulatory T cell isolation kit (Miltenyi Biotec). Carboxyfluorescein succinimidyl ester (CFSE)-labelled CD4^+^CD25^−^ cells were cultured with or without the same number of CD4^+^CD25^+^ cells. The cells were stimulated using anti-CD3/CD28 antibody beads (Life Technologies, Rockville, MD, USA) for 96 h, and cell proliferation was analysed by flow cytometry.

### Cell transfer

Splenocytes from WT, PD-1 KO, T-bet Tg and P/T mice were isolated and single-cell suspensions were prepared in PBS. Splenic cells (1·0 × 10^7^) were injected intravenously into the recipient adult Rag-2 KO mice. In other experiments, the CD4^+^CD25^+^ T_regs_ were isolated from splenocytes of WT mice using CD4^+^CD25^+^ T_reg_ isolation kit (Miltenyi Biotec). The purity of all populations was determined with FACS before injection. Mice were observed every other day for clinical signs of autoimmune syndromes. Histological determination of autoimmune responses against various organs and tissues was conducted at 10 weeks after transfer.

### Measurement of serum autoantibodies of sera

Sera were obtained from mice at 6–10 weeks of age. The levels of anti-nuclear antibody (ANA) in serum were measured using the HEPANA test (MBL) and the indirect immunofluorescence assay to analyse the primary reaction (reacting in the antigen fixation slide and sample) and the second-order reaction [reacting in the fluorescein isothiocyanate (FITC)-labelled antibody]. The levels of anti-double-stranded (ds)DNA antibody in serum were measured by using the anti-dsDNA mouse ELISA kit (Shibayagi, Japan); ELISA to analyse the primary reaction (reacting in the antigen fixation well and diluted sample), the second-order reaction (reacting in the peroxidase binding anti-mouse IgG antibody) and tertiary reaction (reacting in the TMB).

### Statistical analysis

Data were expressed as mean ± standard error of the mean (s.e.m.). Differences between groups were examined for statistical significance using the Mann–Whitney *U*-test except for the survival rate, which was examined using the Wilcoxon–Breslow test. *P*-values less than 0·05 were considered significant.

## Results

### Over-expression of T-bet shortens the life span of PD-1 KO mice

**Figure 1:**
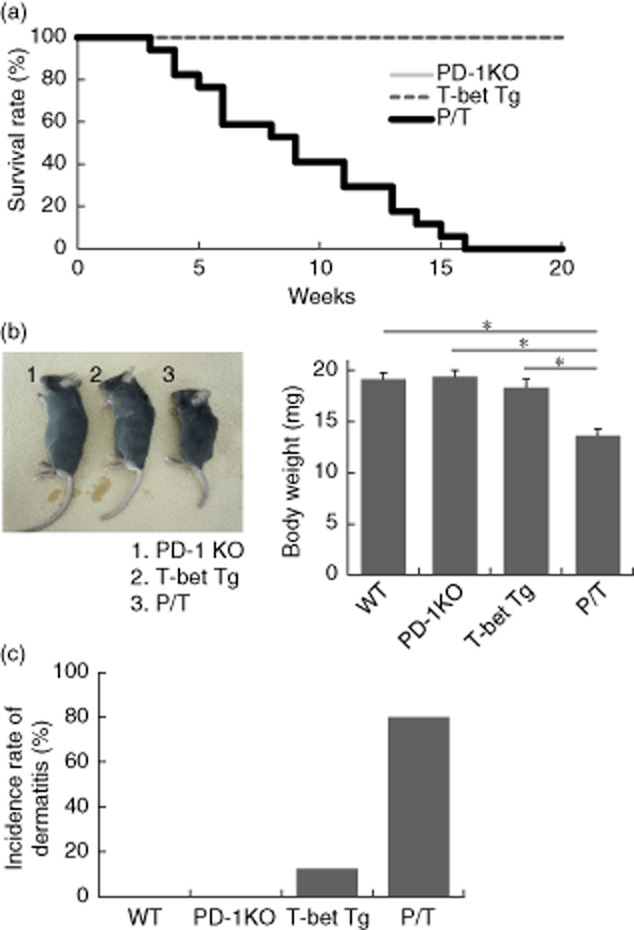
Effects of T-bet over-expression in programmed cell death-1 knock-out (PD-1 KO) mice. (a) Kaplan–Meier curves represent survival rate in the three groups [PD-1 KO mice (*n* = 8), T-bet transgenic (Tg) mice (*n* = 8) and PD-1-deficient T cell-specific T-bet Tg (P/T) (*n* = 17) mice]. Statistical significance was determined using the Wilcoxon–Breslow test. *P* < 0·0005. (b) The growth state was considered by macroscopic appearance and body weight on 5 weeks of life in WT, PD-1 KO, T-bet Tg and P/T mice. Data are mean ± standard error of the mean of five to eight mice per group. (c) The bar graph indicates an incidence rate of dermatitis in WT, PD-1 KO, T-bet Tg and P/T mice. Two-tailed unpaired *U*-test was used for all statistical analyses; *P < 0·05. P/T mice, littermate PD-1 KO mice and T-bet Tg mice were bred under specific pathogen-free conditions.

To examine the effect of T-bet over-expression in PD-1 KO mice, we generated T-bet over-expressing PD-1 KO mice by crossing T-bet transgenic (T-bet Tg) mice under the promoter of CD2 gene with PD-1 KO mice (PD-1 KO × T-bet Tg mice; P/T mice). Surprisingly, the survival rate was significantly lower in P/T mice than littermate PD-1 KO mice (Fig. 1a). The majority of P/T mice, but not littermate PD-1 KO mice, died within 10 weeks (*P* < 0·0005). In the P/T mice the median survival time was approximately 9 weeks, and the mortality rate at 10 weeks was 58·8%. Moreover, body weight was lower and the incidence rate of dermatitis was higher in P/T mice compared with WT, PD-1 KO and T-bet Tg mice at 5 weeks of age (Fig. 1b,c). These observations suggested systemic disorder(s) in P/T mice that negatively affected their life span.

### PD-1 deficiency in T-bet Tg mice induces hepatitis, pancreatitis and enteritis

**Figure 2:**
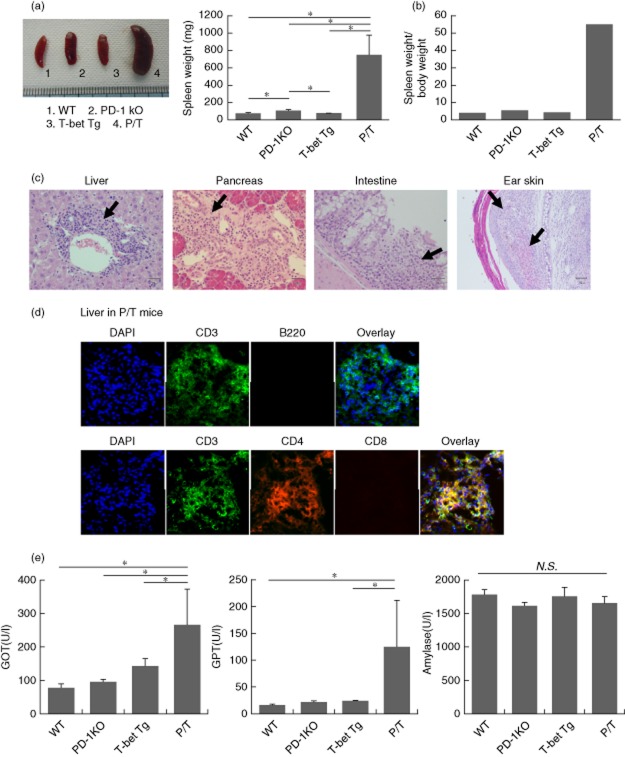
Hepatitis induced by programmed cell death-1 (PD-1) deficiency in T-bet transgenic (Tg) mice. (a) The state of spleen in wild-type (WT), PD-1 knock-out (KO), T-bet Tg and PD-1-deficient T cell-specific T-bet Tg (P/T) mice was showed by gross appearance, spleen weight (*n* = 6 for all groups). (b) The histogram of spleen weight (average)/body weight (average) was derived from computational investigation. (c) Liver, pancreas, intestine and skin of P/T mice of 5-week-old animals were processed for haematoxylin and eosin staining. Arrows indicate leucocyte infiltration. At least four mice were examined from each strain. (d) Infiltrating cells in liver were assessed by immunohistochemistry using anti-CD3 antibody (green), anti-B220 (red), anti-CD4 antibody (red) or anti-CD8 antibody (red) (*n* = 6 for all groups). (e) Serological tests were conducted of glutamic oxaloacetic transaminase (GOT), glutamic pyruvic transaminase (GPT) and amylase at 6–10 weeks of age in WT, PD-1 KO, T-bet Tg and P/T mice. Data are mean ± standard deviation of the mean of five to seven mice per group. The two-tailed unpaired *U*-test was used in all statistical analyses. *P < 0·05.

To determine the cause of death in P/T mice, we examined various body organs macroscopically and histologically. The organs examined were the kidney, heart, spleen, lung, liver, pancreas, salivary gland, lacrimal gland, intestine, mesenteric lymph node (LN) and ear (Table 1). In P/T mice the spleen was swollen, with weight significantly higher than WT mice, PD-1 KO mice and T-bet Tg mice (Fig. 2a,b). Histological evaluation showed infiltration of mononuclear cells in liver, pancreas, intestine and skin in P/T mice (Fig. 2c and Supporting information,Fig. S1). Immunohistochemical analysis indicated that the infiltrated cells in liver were CD3^+^CD4^+^ cells (Fig. 2d). Serological tests were conducted at 6–10 weeks of age to evaluate the severity of organ dysfunction. Serum levels of GOT and GPT were significantly higher in P/T mice than in PD-1 KO mice (Fig. 2e). However, serum amylase levels were similar in WT, PD-1 KO, T-bet Tg and P/T mice. These findings suggest that PD-1 deficiency in T-bet Tg mice induced systemic inflammation and liver damage.

**Table 1:** Histological analyses in organs

	Kidney	Heart	Spleen	Lung	Liver	Pancreas	Salivary gland	Lacrimal gland	Intestine	Mesenteric LN	Ear skin
WT	−	−	−	−	−	−	−	−	−	−	−
PD-1 KO	−	−	−	−	−	−	−	−	−	−	−
T-bet Tg	−	−	−	−	−	−	−	−	−	−	−
P/T	−	−	−	−	+	+	−	−	+	−	+

The organs were isolated from wild-type (WT), programmed cell death-1 knock-out (PD-1 KO), T-bet transgenic (Tg) and PD-1-deficient T cell-specific T-bet Tg (P/T) mice at 4–8 weeks old. Histological analyses were performed with haematoxylin and eosin (H&E) stain. At least five mice were examined from each strain. Mean histological score on a two-point scale; (−) denotes no infiltrates, (+) denotes inflammation.

### Over-expression of T-bet and high IFN-γ production by splenic CD4^+^T cells of P/T mice

**Figure 3:**
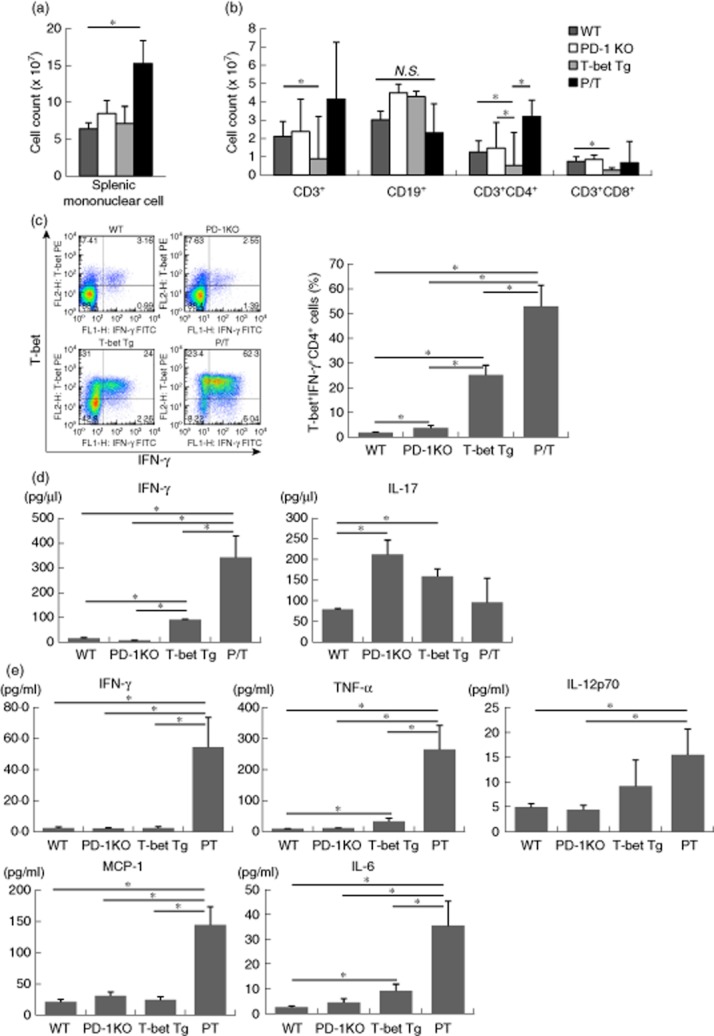
Concomitant over-expression of T-bet and interferon (IFN)-γ in peripheral T cells. (a) The number of splenic mononuclear cells counted with the use of a haemocytometer. (b) CD3^+^, CD19^+^, CD3^+^CD4^+^ or CD3^+^CD8^+^ cells in spleen analysed by flow cytometry, and cell number was calculated by data. All data are mean ± standard deviation of the mean (s.e.m.) of four to five mice per group. (c) CD4^+^ T cells were stimulated with phorbol myristate acetate (PMA)/ionomycin for 6 h. T-bet expression and IFN-γ production by CD4^+^ T cells were analysed by intracellular staining. All data are mean ± s.e.m. of four to five mice per group. (d) CD4^+^ T cells were cultured with anti-CD3/CD28 antibody, and IFN-γ and interleukin (IL)-17 levels in culture supernatants were analysed by enzyme-linked immunosorbent assay (ELISA). The two-tailed unpaired *U*-test was used for statistical analysis. *P < 0·05. (e) Serum levels of cytokine were determined by cytometric bead array (CBA). Means ± s.e.m. (*n* = seven to 11 mice per group). The two-tailed unpaired *U*-test was used for all statistical analyses. *P < 0·05.

The above results suggested that systemic inflammation was induced by T-bet over-expressing T cells in P/T mice. In the next step, we examined lymphocyte proportions and T cell activation in peripheral blood. Although the splenic mononuclear cell count was higher in P/T mice than in WT mice, FACS analysis of lymphocytes in the spleen showed similar CD3^+^CD4^+^ cell counts in WT and PD-1 KO mice (Fig. 3a,b). We next analysed cytokine production and transcription factor expression on CD4^+^ T cells in spleen by FACS. Intracellular cytokine staining of PMA/ionomycin-stimulated splenocytes showed over-expression of T-bet and higher IFN-γ production on CD4^+^ T cells in P/T mice (52·85 ± 8·57%) compared to WT (1·89 ± 0·18%), PD-1 KO (3·76 ± 1·00%) and T-bet Tg mice (25·13 ± 3·90%) (Fig. 3c). In the next set of experiments, CD4^+^ T cells isolated from the spleen were stimulated with anti-CD3 monoclonal antibody (mAb) and anti-CD28 mAb *in vitro* for 48 h, and cytokine levels in the culture supernatants were analysed using ELISA. IFN-γ was significantly higher in P/T mice than WT, PD-1 KO and T-bet Tg mice, while IL-17 was comparable in the four groups (Fig. 3d). To identify the cause of systemic inflammation in P/T mice, we measured proinflammatory cytokine levels in sera by CBA. IFN-γ, TNF-α, IL-12p70, MCP-1 and IL-6 were elevated significantly in P/T mice compared with WT, PD-1 KO and T-bet Tg mice (Fig. 3e). Moreover, we measured serum autoantibodies, but ANA and anti-dsDNA antibodies were not detected in sera of P/T mice (data not shown). These results suggest that PD-1 suppresses T-bet expression, IFN-γ and the production of various inflammatory cytokines.

### Low percentage of FoxP3^+^ regulatory T cells in P/T mice

**Figure 4:**
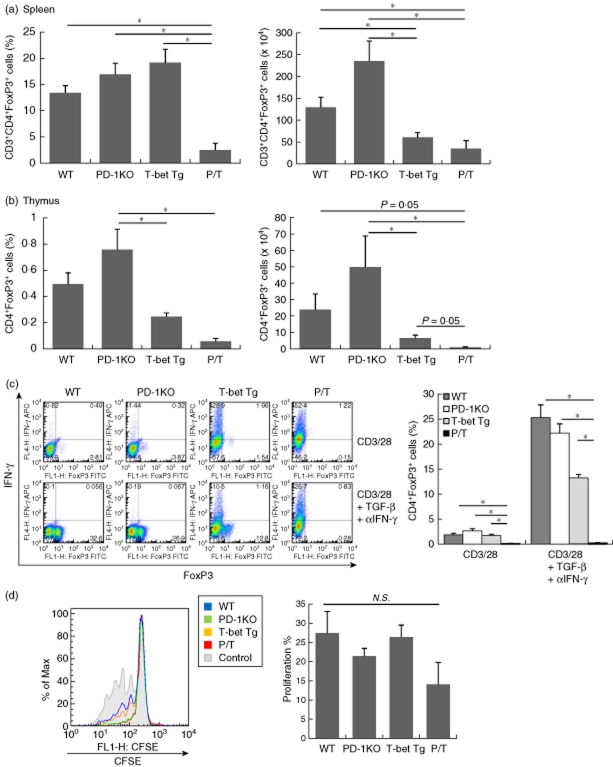
Regulatory T cell (T_reg_) differentiation and function *in vivo*. (a, b) Percentage of forkhead box protein 3 (FoxP3)^+^ cells among CD4^+^ T cells and number of FoxP3^+^CD4^+^ T cells were analysed by intracellular staining in spleen (a) and thymus (b). Data are representative of three independent experiments with four or more mice in each group and are shown as the mean ± standard deviation of the mean (s.e.m.). (c) CD4^+^ T cells were cultured with transforming growth factor (TGF)-β and anti-interferon (IFN)-γ in the presence of interleukin (IL)-2. Five days later, T cell FoxP3 and IFN-γ expression were analysed by flow cytometry. Data are representative of three independent experiments with triplicate culture wells and are shown as the mean ± s.e.m. (d) T_reg_ suppression assay showing carboxyfluorescein succinimidyl ester (CFSE) proliferation profiles of cultured CD4^+^ T cells, CD3-specific monoclonal antibody and untreated T_regs_ (ratios are T_reg_ : effector T cells = 1:1). Numbers represent CFSE-labelled CD4^+^ T cells after 72 h in culture. Data are representative of three independent experiments with triplicate culture wells and are shown as the mean ± s.e.m. The two-tailed unpaired *U*-test was used for statistical analysis. *P < 0·05.

To examine the role of T-bet over-expression in the development of T_regs_ in spleen and thymus, we analysed splenocytes and thymocytes from P/T mice. FACS analysis of the spleen showed that the percentage of FoxP3^+^ cells among CD4^+^ T cells (FoxP3^+^ T_regs_) was significantly lower in P/T mice than WT, PD-1 KO and T-bet Tg mice, and that the number of FoxP3^+^ T_regs_ was lower in T-bet Tg and P/T mice than in WT and PD-1 KO mice (Fig. 4a). In the thymus, the number of FoxP3^+^ T_regs_ was also lower in P/T mice than in WT and PD-1 KO mice (Fig. 4b). To confirm the ability of CD4^+^ T cells to differentiate into FoxP3^+^ T_regs_, CD4^+^ T cells isolated from WT, PD-1 KO, T-bet Tg and P/T mice were cultured in the presence of TGF-β and anti-IFN-γ mAb, and FoxP3 and IFN-γ expression was determined after 5-day culture by flow cytometry. TGF-β induced FoxP3 expression in CD4^+^ T cells of WT, PD-1 KO and T-bet Tg mice, but not P/T mice (Fig. 4c). FoxP3^+^ T_reg_ function was analysed by the culture of CD4^+^CD25^−^ effector cells with or without CD4^+^CD25^+^ (T_reg_) cells. We confirmed that there was no significant difference in FoxP3 expression in CD4^+^CD25^+^ T_regs_ isolated from WT, PD-1 KO, T-bet Tg and P/T mice (Supporting information,Fig.S2). The suppression of effector T cell proliferation by P/T mice-derived T_regs_ was comparable with WT, PD-1 KO and T-bet Tg mice-derived T_regs_ (Fig. 4d). The P/T mice-derived T_regs_ had a greater tendency to suppression of effector T cell proliferation than other mice-derived T_regs_, but showed no significant difference. The above results suggested suppression of differentiation and/or maintenance of T_regs_ in peripheral blood of P/T mice.

### Co-injection of WT T_regs_ and P/T cells partially suppresses systemic inflammation in Rag-2 KO mice

**Figure 5:**
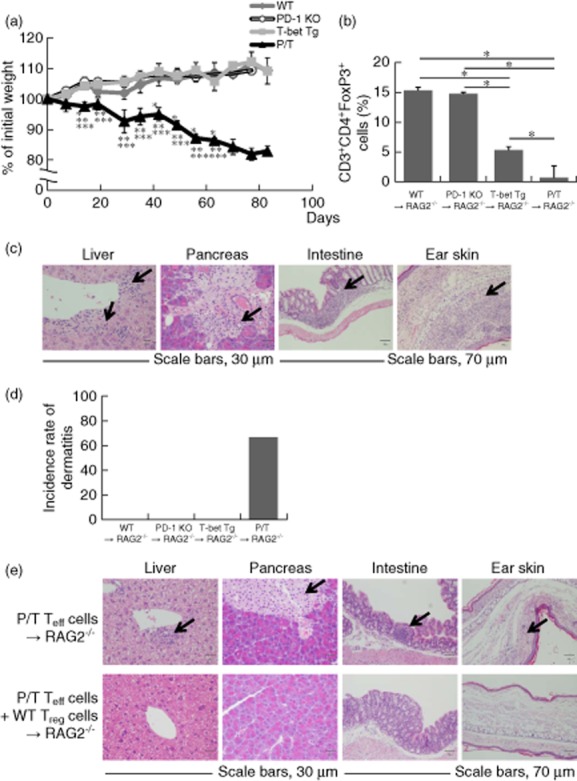
Transfer of forkhead box protein 3 (FoxP3)^+^ regulatory T cells (T_regs_) from wild-type (WT) mice reduces inflammation in recipient mice. (a–d) Adult recombination activating gene 2 (Rag-2) knock-out (KO) mice were injected intravenously with 1 × 10^7^ splenocytes from WT, programmed cell death-1 knock-out (PD-1 KO), T-bet transgenic (Tg) or PD-1-deficient T cell-specific T-bet Tg (P/T) mice. (a) Relative body weight of recipient mice injected with splenocytes (*n* = 6 for all groups). Statistical significance was determined using Student's *t*-test. *P* < 0·05 (*WT mice *versus* P/T mice; **PD-1 KO mice *versus* P/T mice; ***T-bet Tg mice *versus* P/T mice). (b) Adult Rag-2 KO mice were injected intravenously with splenocytes from WT, PD-1 KO, T-bet Tg or P/T mice. The percentage of FoxP3^+^ CD4^+^ T cells was analysed by intracellular staining in spleen 10 weeks after injection. Data are representative of three independent experiments with four or more mice in each group and are shown as the mean ± standard deviation of the mean. Two-tailed unpaired *u*-test was used for statistical analysis. *P < 0·05. (c) Ten weeks later, the histological slides of liver, pancreas, intestine and skin were examined for inflammation by haematoxylin and eosin (H&E) staining. Arrows indicate leucocyte infiltration. At least five mice were examined from each strain. (d) The bar graph indicates an incidence rate of dermatitis. (e) Adult Rag-2 KO mice were injected intravenously with 1 × 10^6^ P/T CD4^+^CD25^−^ cells with or without 1 × 10^6^ CD4^+^CD25^+^ cells (T_regs_) from WT mice. The histological slides of liver, pancreas, intestine and skin after 10 weeks were examined for inflammation by H&E staining. Arrows indicate leucocyte infiltration. At least five mice were examined from each strain.

To confirm the pathogenic mechanisms in P/T mice, splenocytes of P/T mice were injected into Rag-2 KO mice. Although this transfer did not result in death of the recipient mice within the observation period, it resulted in a significant drop in body weight compared with mice that received splenocytes from WT, PD-1 KO or T-bet Tg mice (Fig. 5a). Because P/T mice showed a significant reduction of FoxP3^+^ T_regs_, we performed FACS analyses of splenocytes from recipient Rag-2 KO mice. The percentage of FoxP3^+^ cells in CD4^+^ cells was lower in recipient mice injected with splenocytes of P/T mice compared with WT, PD-1 KO and T-bet Tg mice (Fig. 5b). Histological analysis at 10 weeks after the transfer of splenocytes showed infiltration of mononuclear cells in the liver, pancreas, intestine and skin of recipient Rag-2 KO mice, similar to P/T mice, but no such infiltration was noted in Rag-2 KO mice recipient of splenocytes from WT, PD-1 KO and T-bet Tg mice (Table 2 and Fig. 5c). Moreover, the dermatitis was developed only in recipient mice transfer with splenocytes from P/T mice (Fig. 5d). Next, we examined the effects of CD4^+^ T cells of P/T mice on the development of inflammatory symptoms in Rag-2 KO mice by injecting CD4^+^CD25^−^ cells from P/T mice into Rag-2 KO mice. Histological analysis showed infiltration of mononuclear cells, similar to that seen following the injection of splenocytes from P/T mice into Rag-2 KO mice (Fig. 5e). To determine whether the reduction of FoxP3^+^ T_regs_ was related to mononuclear cell infiltration in multiple organs in recipient mice transferred with cells of P/T mice, we injected CD4^+^CD25^−^ cells from P/T mice into Rag-2 KO mice with or without CD4^+^CD25^+^ cells isolated from WT mice. No mononuclear cell infiltration was noted in the liver, pancreas, intestine and skin of Rag-2 KO mice injected with P/T mice and CD4^+^CD25^+^ cells isolated from WT mice (Table 3 and Fig. 5e). These results indicated that the presence of low percentages of T_regs_ of P/T mice induced systemic inflammation and body weight loss in recipient Rag-2 KO mice.

**Table 2:** Histological comparison of organ inflammation among that recombination activating gene 2 (RAG-2) knock-out (KO) mice injected with programmed cell death-1 (PD-1)-deficient T cell-specific T-bet transgenic (P/T) splenocytes

		Kidney	Heart	Spleen	Lung	Liver	Pancreas	Salivary gland	Lacrimal gland	Instestine	Mesenteric LN	Ear skin
Rag-2 KO	WT splenocytes	−	−	−	−	−	−	−	−	−	−	−
	PD-1 KO splenocytes	−	−	−	−	−	−	−	−	−	−	−
	T-bet Tg splenocytes	−	−	−	−	−	−	−	−	−	−	−
	P/T splenocytes	−	−	−	−	+	+	−	−	+	−	+

Adult Rag-2 KO mice were injected intravenously with 1 × 10^7^ splenocytes. Ten weeks later, their organs were analysed histologically for inflammation. At least four mice were examined from each strain. Mean histological score on a two-point scale; (−) denotes no infiltrates, (+) denotes inflammation.

**Table 3:** Effects of wild-type (WT) regulatory T cells (T_regs_) on inflammation in recombination activating gene 2 (RAG-2) knock-out (KO) mice injected with programmed cell death-1 (PD-1)-deficient T cell-specific T-bet transgenic (P/T) effecter T cells (T_eff_)

		Kidney	Heart	Spleen	Lung	Liver	Pancreas	Salivary gland	Lacrimal gland	Instestine	Mesenteric LN	Ear skin
Rag-2 KO	P/T T_eff_	−	−	−	−	+	+	−	−	+	−	+
	P/T T_eff_ + WT T_reg_	−	−	−	−	−	−	−	−	−	−	−

Adult Rag-2 KO mice were injected intravenously with 1 × 10^6^ P/T CD4^+^CD25^−^ cells (effector T cells : T_eff_ cells) alone or with 1 × 10^6^ CD4^+^CD25^+^ T cells (T_reg_ cells). Ten weeks later, their organs were analysed histologically for inflammation. At least four mice were examined from each strain. Mean histological score on a two-point scale; (−) denotes no infiltrates, (+) denotes inflammation. LN = lymph nodes.

## Discussion

In the present study, we generated PD-1 deficient T-bet Tg (P/T) mice to examine the role of PD-1 on the differentiation and function of Th1 cells both *in vivo* and *in vitro*. P/T mice showed growth retardation, and the majority died within 10 weeks. They also showed severe splenomegaly and dermatitis and histological evidence of hepatitis, pancreatitis and colitis. Splenomegaly was reflected in the increase of splenic mononuclear cells in P/T mice. Moreover, FACS analysis of splenocytes demonstrated the presence of large proportions of T-bet^+^ IFN-γ^+^ Th1 cells among CD4^+^ T cells and also a markedly low proportion of FoxP3^+^ T_regs_ in the spleen of P/T mice, and the various inflammatory cytokines by CBA analysis were elevated significantly in sera. Based on the above finding, we propose that the abnormal balance between Th1 cells and Foxp3^+^ T_regs_ played a major role in the induction of systemic inflammation in P/T mice.

Ishizaki . 31 reported that T-bet Tg mice developed dermatitis characterized by swollen, flaky and scaly skin in regions without body hair, which is pathologically similar to that seen in contact dermatitis, and that CD4^+^ T cells differentiated mainly into Th1 cells. Moreover, spontaneous development of characteristic lupus-like mild proliferative glomerulonephritis with predominant IgG3 deposition was reported in ageing C57BL/6 PD-1 KO mice, together with the appearance of arthritis characterized by increased lining layers of synovial tissues in foot joints 23. Although we confirmed a slight but significant increase in Th1 cells in T-bet Tg mice, pathological assessment showed no evidence of infiltration of mononuclear cells in organs except in the ear skin in T-bet Tg mice, and no death of either T-bet Tg or PD-1 KO mice was observed within the study observation period. These findings indicate that systemic inflammation observed in P/T mice did not result solely from over-expression of T-bet or PD-1 deficiency alone.

The percentage of FoxP3^+^ T_regs_ was clearly low in P/T mice. FoxP3 deficiency induces tissue inflammation and early death, as reported in scurfy mice, which lack CD4^+^CD25^+^FoxP3^+^ T_regs_ 33–35. Scurfy mice are X-linked recessive mice mutants, and all hemizygous male mice with this abnormality die 3–5 weeks after birth. They exhibit over-proliferation of CD4^+^ T lymphocytes, high serum levels of numerous cytokines and extensive infiltration of liver, pancreas and ear skin 35–37, which is roughly similar to the findings in P/T mice. Therefore, we speculate that the short life span and systemic inflammation observed in P/T mice are due to the increase in Th1 cells and reduction in FoxP3^+^ T_regs_. Previous studies showed that adoptive cell transfer of lymph node cells isolated from scurfy mice into Rag-1 KO mice induced systemic inflammation resembling the phenotype of scurfy mice, that cell transfer of neonatal thymectomy (NTx)-PD-1 KO mice into Rag-2 KO mice induced hepatitis and that adoptive cell transfer of Th1 cells into Rag-2 KO mice induced colitis, and such inflammation was suppressed by co-transfer of FoxP3^+^ T_regs_ 37–42. We also used adoptive transfer of splenocytes from WT, PD-1 KO mice, T-bet Tg mice and P/T mice into Rag-2 KO mice to clarify whether the increase of Th1 cells and the reduction of FoxP3^+^ T_regs_ is the cause of the shortened life span and systemic inflammation in P/T mice. Splenocytes of P/T mice induced body weight loss and mononuclear cell infiltration in liver, pancreas, colon and skin in recipient Rag-2 KO mice. Moreover, no fall in body weight was noted and histological findings were attenuated in Rag-2 KO mice when co-transferred with CD4^+^CD25^−^ cells of P/T mice and CD4^+^CD25^+^ T_regs_ from WT mice. These findings support the notion that a low FoxP3^+^ T_reg_ percentage is a major factor in systemic inflammation in P/T mice.

Why is the FoxP3^+^ T_reg_ percentage low in P/T mice? Our results of *in-vitro* induction of FoxP3^+^ T_regs_ demonstrated that FoxP3 expression was not induced in CD4^+^ T cells in P/T mice, suggesting that peripheral CD4^+^ T cells did not differentiate into FoxP3^+^ T_regs_ in P/T mice. Although our results did not define the exact mechanism of the low FoxP3^+^ T_reg_ percentage, we propose the following three possibilities. First, PD-1 deficiency might affect the development and maintenance of induced T_regs_ (iT_regs_), because PD-1/PD-1 ligand interaction plays a pivotal role in the development, maintenance and function of iT_regs_ 43, and PD-L1 and PD-L2 double KO mice show significantly reduced conversion of iT_regs_ and rapidly develop fatal inflammation 44. Secondly, abundant production of IFN-γ might suppress the differentiation of FoxP3^+^ T_regs_, as IFN-γ is a negative regulator of the induction of FoxP3^+^ T_regs_ 45,46. The past study showed that IFN-γ-producing Th1 cells are resistant to the inhibition of de-differentiation into FoxP3^+^ T_regs_ by signal transducer and activator of transcription-1 (STAT-1)-phosphorylation through the activation of STAT-1 34 *in vivo* and *in vitro* 47. In fact, CD4^+^ T cells in P/T mice produced large amounts of IFN-γ under conditions that favoured T_reg_ differentiation *in vitro*. Thirdly, over-expression of T-bet in CD4^+^ T cells could directly inhibit the expression of FoxP3, because Th1 polarization is reported to underlie the pathogenesis of intestinal inflammation 24–26. Although the above scenarios should be investigated in more detail in the future, one of these factors alone does not seem sufficient to explain the low FoxP3^+^ T_reg_ percentage in P/T mice, because no significant difference was observed in the percentage of FoxP3^+^ T_regs_ in splenic CD4^+^ T cells among WT, PD-1 KO and T-bet Tg mice. Moreover, PD-1 deficiency in T-bet Tg mice enhanced the expression of T-bet and production of IFN-γ. Thus, we considered that PD-1 deficiency, high production of IFN-γ and T-bet over-expression together regulate the development and maintenance of FoxP3^+^ T_regs_ in P/T mice.

In conclusion, PD-1 deficiency in T-bet Tg mice caused systemic inflammation and early death, which was probably induced by the augmented Th1 response and low FoxP3^+^CD4^+^ T_reg_ percentage. Therefore, we consider that the cause of death in P/T mice is multiple organ damage resulting from severe inflammation. These findings could be helpful for understanding the molecular mechanism of Th1-mediated diseases and development of Th1 cell targeting therapy.

## Supporting Information

**Fig. S1.** PD-1-deficient T cell-specific T-bet transgenic (Tg) (P/T) mice developed lymphocytic infiltration in liver, pancreas, intestine and skin. Liver, pancreas, intestine and skin of wild-type (WT), programmed death-1 knock-out (PD-1 KO), T-bet Tg and P/T mice of 5-week-old animals were processed for haematoxylin and eosin (H&E) staining. At least four mice were examined from each strain. **Fig. S2.** The percentage of CD4^+^CD25^+^forkhead box protein 3 (FoxP)3^+^ cells were not different. Percentage of FoxP3 expression on CD4^+^CD25^+^ T cells were analysed by intracellular staining in spleen. Data are representative of three independent experiments with four or more mice in each group and are shown as the mean ± standard error of the mean.
