# Supplementary material for: T-bet regulates differentiation of forkhead box protein 3+ regulatory T cells in programmed cell death-1-deficient mice
Source: Clin Exp Immunol. 2015 Jan 2;179(2):197–209. doi: 10.1111/cei.12455 (PMC4298397; doi:10.1111/cei.12455)
Supplement: Supplementary file 1 — Fig. S1. PD-1-deficient T cell-specific T-bet transgenic (Tg) (P/T) mice developed lymphocytic infiltration in liver, pancreas, intestine and skin. Liver, pancreas, intestine and skin of wild-type (WT), programmed death-1 knock-out (PD-1 KO), T-bet Tg and P/T mice of 5-week-old animals were processed for haematoxylin and eosin (H&E) staining. At least four mice were examined from each strain. Fig. S2. The percentage of CD4+CD25+forkhead box protein 3 (FoxP)3+ cells were not different. Percentage of FoxP3 expression on CD4+CD25+ T cells were analysed by intracellular staining in spleen. Data are representative of three independent experiments with four or more mice in each group and are shown as the mean ± standard error of the mean. [file cei0179-0197-sd1.pptx]

## Slide 1
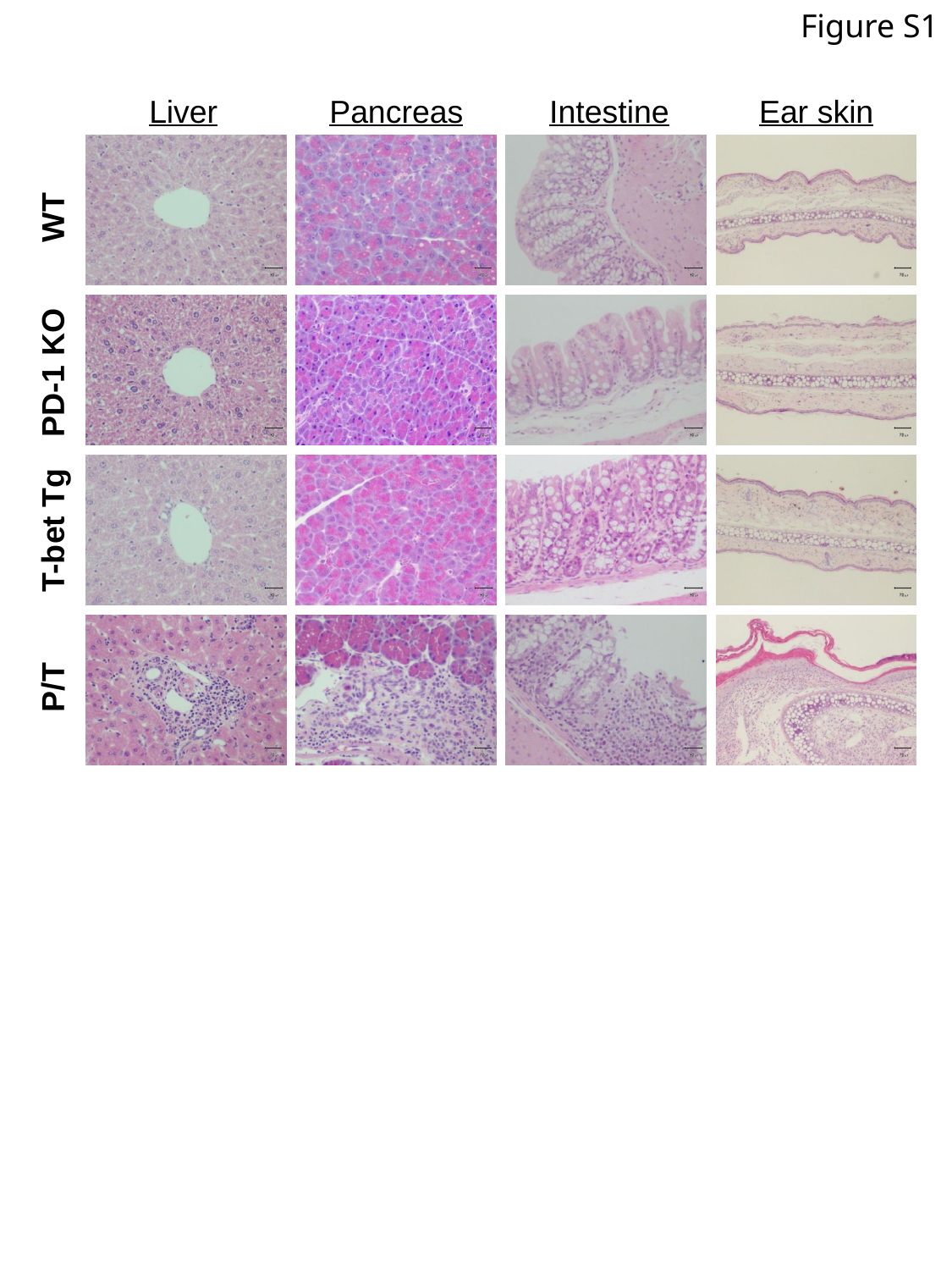

Figure S1
Liver
Pancreas
Intestine
Ear skin
WT
PD-1 KO
T-bet Tg
P/T

## Slide 2
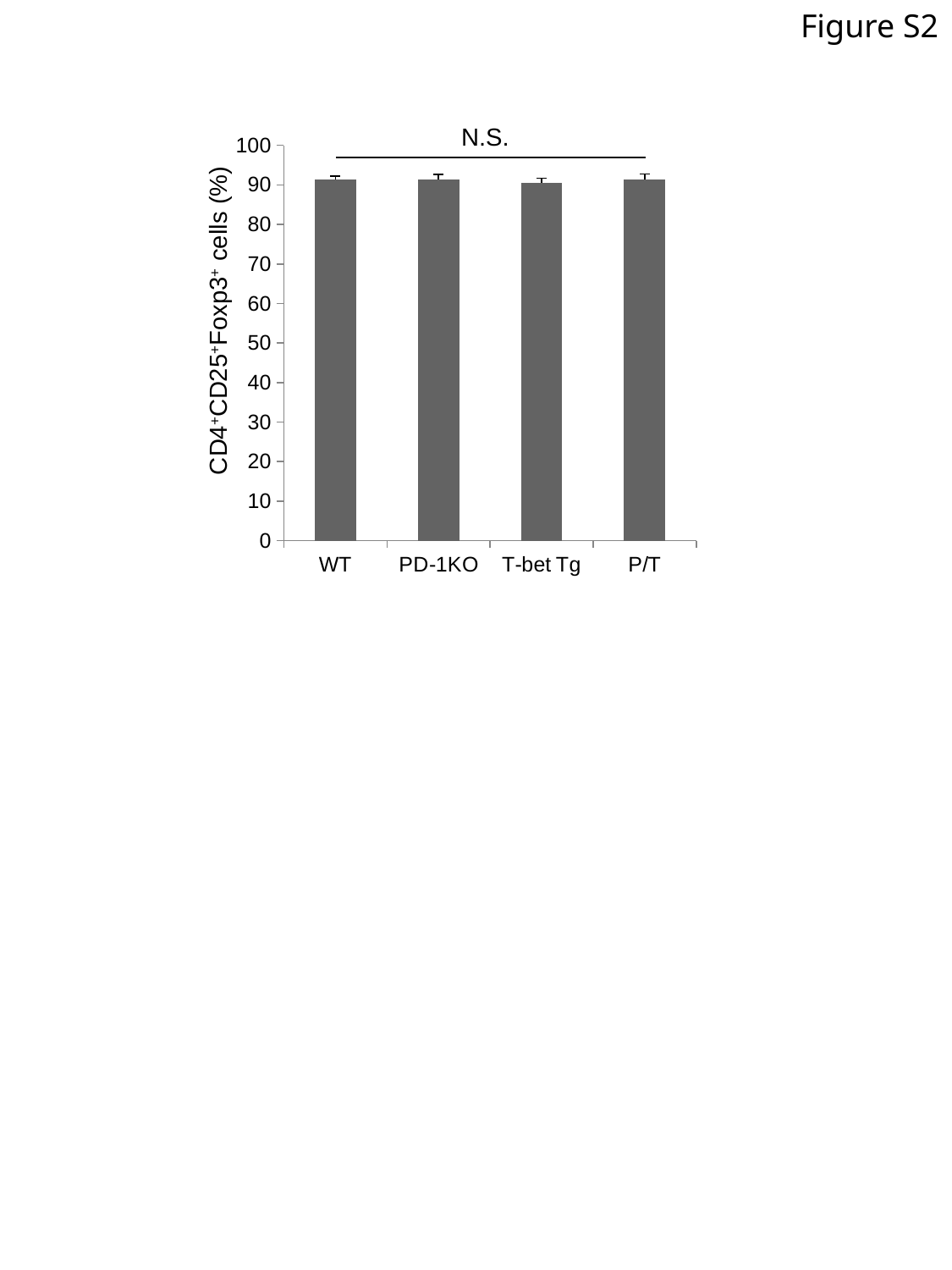

Figure S2
N.S.
### Chart
| Category | |
|---|---|
| WT | 91.30000000000001 |
| PD-1KO | 91.3 |
| T-bet Tg | 90.52499999999999 |
| P/T | 91.325 |CD4+CD25+Foxp3+ cells (%)
